# Notices for May

**Published:** 1873-05

**Authors:** 


### NOTICES FOR MAY.

HARPER'S HAND-BOOK for travelers in Europe, for 1873, of near 800 pages, with 86 maps and plans of cities, contains a rich fund of information about everything an intelligent traveler wishes to know in reference to all places of historical interest, the distance between places, routes, and prices of conveyance, the principal hotels and prices, first and second class. Price $5. It is the best book of the kind ever published in America, for it is full and accurate.

SUMMER RECREATION.—For sea-side resort, Narragansett Pier is the most desirable in America, and on many accounts ; it is on the shore of the broad Atlantic, the nearest land being Ireland. The beach is sandy, of a gentle declivity, no undertow, and no bather has ever lost a life there. This beach is within a quarter of a mile of the hotels ; the side-walks reach to within fifty yards of the water-line. The usual charge for rooms in 1872 was from $25 to $30 a week for two persons in a room. The Pier is within ten miles of Newport, across the bay ; a steamer makes the trip several times a day. The New Haven cars at Forty-second street depot take passengers there within eight hours. There are about twenty hotels in the place ; all crowded during the season. The Tower Hill House is back from the water, but horse-cars take the guests to the beach every day, free of charge ; there are 800 acres of pasture and woodland around the hotel for the benefit of the guests ; the air is pure, the scenery, of land and water, is magnificent ; and being away from the noise and dust of the village, gives it a great advantage over the other houses. There are several cottages connected with the hotel, adapted to families. The smaller hotels take families of children at very low rates. Land for building can be had by the foot or acre on the hill and reaching down to the water's edge. There is a handsome Episcopal church, and houses of worship of other churches, in the villages around within four or five miles, with beautiful drives in every direction.

DRUNKENNESS CURED.—Report of the proceedings of the third meeting of the American Association for the Cure of Inebriates, held in New York, Oct. 8–10, 1872, a most useful and suggestive pamphlet of 130 pages, 8vo. JOSEPH PARISH, M. D., President Copies will be sent, postpaid, by sending fifty cents to Thomas A. Boyd, Director at Media, near Philadelphia, Pa., detailing among other things the mode of curing delirium tremens and convulsions, and the prevention of drunkenness, repression and cure of drunkenness, announcement of the places where there are institutions for the cure of the disease, with a very large amount of most useful information in reference to the whole subject. President Parish is a nation's benefactor, for all the head-work he has done, in pushing forward the building of institutions, and in diffusing, by his facile pen, important information in reference to the best means of reclaiming unfortunates.

The Philosophy of the Christian Religion. By T. N. HORNSBY. Published by Davidson Brothers, of Louisville, Ky. Sent, postpaid, for 20 cents. It discusses well, and in a good spirit, the inquiry: When will the general judgment take place, and the body rise, and with what body? The first chapter is under the heading, "Friend, go up higher."

MAISON DE SANTE.—If encouragement is given, it is contemplated to establish an institution in the city of New York for the medical and surgical treatment of constipation, piles and fissure, abscess, fistula, stricture, ulceration, polypoid tumors, malignant degeneration, and the diseases of adjacent parts. There is but one institution of the kind in the world—St. Mark's Hospital, London—and it has been of incalculable service. If Dr. Wm. Boderhemer, 2 West Forty-third street, New York, could be induced to head such an establishment in New York city, a public benefit would result, very far reaching in its good effects, and would be destined to do immeasurable good. There is no physician living who has had such a long and large success in the treatment of the maladies named; he has a national reputation, and has written several exhaustive works on the above diseases. To save us the trouble of answering letters of inquiry, Dr. B.'s address in full is given without consulting him; this article was written in the interest of our subscribers, and not that of publishers.

"Satchel Guide," for travelers going abroad this summer, is for making notes, memoranda, expenses, engagements, etc., etc., with a large amount of useful, in fact, indispensable information about various routes of travel, ship lines, cost, items in reference to the Vienna Exposition. An intelligent, systematic traveler could not spend any two dollars to as great an advantage as in the purchase of this very useful book. Published by Hurd & Houghton.

Memento Mori of the two loved and loving sisters, Alice and Phœbe Carey, with some of their late poems. By MARY CLEMMER AMES. With two steel portraits, 351 pp., 12mo., $1.50. Hurd & Houghton, Publishers. A book which tens of thousands of appreciative readers will peruse with affectionate remembrances.

"The Sanitarian," or Health Maker. A. N. BELL, M. D., Editor. $3 a year. Is a monthly journal published by A. S. Barnes & Co., New York and Chicago, who say, "The purpose of this publication is so to present the results of the various inquiries which have been and which may hereafter be made for the preservation of health and the expectation of human life, as to make them most advantageous to the public and the medical profession." We cordially wish the new comer a wide, useful and remunerative career.

The London "Lancet" is re-published monthly, for $5 a year, by WILLIAM C. HERALD, 52 John street, New York. It is among the leading medical magazines of the world.

Questions of Quarantine. By ALFRED M. CARROLL, M.D. F. Leypoldt, Publisher, 712 Broadway, New York. On the nature and prevention of communicable diseases. The subject is ably handled, and as the summer approaches, it merits the consideration of both physician and people.

Physical Degeneracy. By NATHAN ALLEN, M. D., of Lowell, Mass. Published by Appleton & Co., New York, 41 pages, 12 cents. Dr. Allen has written much and well on this and kindred subjects; and they are of very great general importance.

Centennial Address before the New York Society Library. By THOMAS WARD, M. D. An admirable lecture delivered with great effect before one of the most aristocratic audiences of New York. It was at once amusing and instructive; the hints at the follies of the times, although genially made, indicated great reflection, close observation and deep thought on the part of the learned lecturer. The audience at once voted for the immediate publication of the address, which had just been listened to with the liveliest satisfaction.

A case of anchylosis of the hip joint, of fourteen years' duration, successfully operated upon. By A. G. BEABEE, A. M., M. D., of Chicago. 8 pp., with cuts, 25 cts. The paper is clearly written, and merits the careful reading of every educated practitioner.

Spectrum Analysis Discoveries, being number four of Half-Hour Recitations in Popular Science. DANA ESTES, Editor. Published by Lee & Shepard, Boston and New York. 25 cents; of entrancing interest.

CONVERSATION, our accountability in it: being a sermon to the Congregational Church at Lacon, Illinois, by its pastor, Rev. F. F. WILLIAMS. 25 cents. Spencer Elsworth, Publisher, Lacon, Illinois. The subject is well handled, and merits the thoughtful consideration of all conscientious persons. A grand, good thing would be done if a million copies were distributed among the Christian families of the land.

